# Bibliography

**Published:** 1918-01

**Authors:** 


### BIBLIOGRAPHY

Modern Dentistry. This is the title of a new book by a well known contributor to dental literature, and a scientific student of dentistry for many years. The author's idea seems to be to produce a book that shall set forth, so far as our present knowledge will permit, the principles and practices involved in rescuing humanity from the injurious influences attending diseases of the teeth and their immediate environment. This he does not do in the usual pedagogic way however, for he seems to have elected to exploit his own ideas of certain subjects which he has more carefully studied, rather than to give an orderly consideration of all the correlated subjects involved in a comprehensive study of the diseases of the teeth and their influence on general health. He begins with a consideration of mouth infection and its prevention, inserts a chapter on the study of the physical qualities of enamel and saliva, then one on diagnosis, then one on the violet ray and local treatment by instrumentation, tartar solvents, etc.; and then a whole chapter on vaccine treatments. The sixth chapter is devoted to the treatment of root canals, the seventh, to porcelain, gold and other fillings; the eighth, to the treatment of childrens teeth from gum lancing to orthodontia; the ninth and tenth chapters are devoted to crown and bridge work; the eleventh chapter to a study of the strength and solubilities of cements, and the last chapter, the interpretation of X-ray films. The book is written in Doctor Heads well known style and is very interesting reading. It will be a most valuable contribution to our professional literature as it puts on record in available form the life work of one of our most earnest and devoted students of dental disease problems. It should be most valuable in the library of the progressive students of dental science and art. It will probably not become popular as a school text because it lacks the pedagogic idea of systematic presentation of a definite subject. We are exceedingly glad Dr. Head has made his work accessible to all students as it is sure to become a most useful reference book. The book is of octavo form and is printed on beautiful heavy paper which shows up admirably the more than 300 illustrations. It is published by the W. B. Saunders Co., of Philadelphia, and sells for five dollars.

